# Editorial: Multiple Implications of the Kynurenine Pathway in Inflammatory Diseases: Diagnostic and Therapeutic Applications

**DOI:** 10.3389/fimmu.2022.860867

**Published:** 2022-02-11

**Authors:** Yvette Mándi, Trevor W. Stone, Gilles J. Guillemin, László Vécsei, Richard O. Williams

**Affiliations:** ^1^ Department of Medical Microbiology and Immunobiology, Albert Szent-Györgyi Medical School, University of Szeged, Szeged, Hungary; ^2^ The Kennedy Institute of Rheumatology, NDORMS, University of Oxford, Oxford, United Kingdom; ^3^ Neuroinflammation Group, Macquarie Medical School, Macquarie University, Sydney, NSW, Australia; ^4^ Department of Neurology, Albert Szent-Györgyi Faculty of Medicine, University of Szeged, Szeged, Hungary

**Keywords:** kynurenine, IDO, inflammation, neurological disease, inflammatory diseases, AhR

The kynurenine pathway is responsible for metabolising most of the free tryptophan in mammals. It is activated by infectious agents, inflammatory mediators and stress, which trigger the induction and activity of key enzymes such as indoleamine-2,3-dioxygenase (IDO1), kynurenine-2,3-monooxygenase (KMO) and kynureninase. The biological effects of the various downstream metabolites of the kynurenine pathway have been linked with symptom development and disease progression in a wide range of disorders (1–3) The aim of this Research Topic - which includes 11 original articles and 2 reviews - is to explore the role of the kynurenine pathway and its metabolites in a wide range of diseases of infectious, autoimmune, or neuro-immunological origin.

There is an extensive interaction between the kynurenine pathway and the immune system (4). Rheumatoid arthritis (RA) is one of the most common inflammatory disorders and its treatment has been revolutionised by the introduction of compounds which interfere with the proinflammatory activity of Tumor Necrosis Factor alpha (TNF-α). However, up to half of RA patients have an inadequate response to these drugs, or lose response over time, so that alternative ways of modulating TNF-α levels or receptors are under investigation. Kynurenic acid might be a potential regulator of inflammatory processes in arthritic joints (5). Balog et al demonstrate that a synthetic analogue of kynurenic acid - SZR72 - inhibited TNF-α production in whole blood samples from RA patients while raising levels of TNF-α-stimulated gene 6. The detailed mechanism of these effects should prove interesting, especially the important question of whether the sites of action of SZR72 overlap those of kynurenic acid itself. Answers would be relevant to other work showing that IDO1 activation can reduce the symptoms of experimental arthritis. Indeed systemic administration of the same analogue, as well as kynurenic acid itself, inhibits many of the consequences of pancreatic inflammation and acinar cell damage in an animal model (Balla et al.)

Kynurenic acid analogues represent potential neuroprotective agents in experimental sepsis. Sepsis is defined as a dysregulated host response to infection, which can lead to life-threatening organ failure. The brain is among the potentially injured vital organs, causing central nervous system (CNS) dysfunctions (6). Poles et al. reported reduced peripheral Neutrophil Extracellular Trap (NET) formation, lowered blood-brain barrier (BBB) permeability changes and alleviation of mitochondrial dysfunction in the CNS by exogenous kynurenic acid or its synthetic analogues SZR-72 and SZR-104 in a clinically relevant rodent model of intra-abdominal sepsis.

TNF-α may also be relevant in other conditions where there is kynurenine pathway activation. In addition to infections of the nervous system, the kynurenine pathway is activated by various forms of stress. Myint et al. report that childhood stressors also increase serum TNF-α levels in parallel with an increased ratio of 3-hydroxyanthranilic acid to anthranilic acid, a ratio previously shown to reflect the presence of inflammation (7) supporting possible links between the kynurenine pathway and TNF-α.

The kynurenine pathway represents a major link between the immune system and other organs especially the CNS (8). The kynurenine pathway generates metabolites, such as quinolinic acid produced by activated monocytic cells, which activate glutamate receptors sensitive to N-methyl-D-aspartate (NMDA) and kynurenic acid, produced by astrocytes, which blocks glutamate receptors. As glutamate is the dominant excitatory neurotransmitter in the CNS, changes in the kynurenine pathway activity can have profound effects on neural function, behaviour, and neurodegeneration. Del’Arco et al. have studied the impact of infection by the parasite *Neospora* and show the associated changes in kynurenine pathway metabolites and their relationship with neurodegeneration.

The problems of understanding kynurenine pathway activity in the CNS relate to the complexity of several interacting factors. Firstly, there is the balance between quinolinic acid as an excitant and potentially neurotoxic compound and kynurenic acid which inhibits neural excitation and is neuroprotective. Secondly, there is the concentration dependence of responses to these agents since excitation at low levels can cause inactivation or become toxic at higher levels. Thirdly, there is a question of how changes in the production of any of the kynurenine metabolites may modify activity in a different cell type or tissue, generating a variety of effects and modulatory influences on other tissues.

During fungal infection by *Paracocidioides*, kynurenine acted on Aryl Hydrocarbon Receptors (AhR) to modulate the production of immune cell sub-populations? (de Araújo et al.) AhR mediate some effects of kynurenine and kynurenic acid on cell growth and differentiation, and represent a central feature of the IDO1-kynurenine-AhR-IDO1 positive feedback system (9, 10). The kynurenines (particularly kynurenine itself) are well known to promote regulatory T cell differentiation, while suppressing pro-inflammatory Th17 cell generation and this concept was extended by the authors to account for the reduced numbers of inflammatory activated CD11c+ cells in the lungs, thus inhibiting pulmonary inflammation. Kynurenine pathway activation is also likely to be involved in other peripheral pathologies such as those affecting the general vascular system (Ramprasath et al.) and coronary arteries (Gáspár et al.).

One of the continuing debates in the field of the research on the kynurenine pathway in the nervous system is whether measurements of kynurenine and its downstream metabolites in peripheral blood reflect their levels within the CNS. Only kynurenine and 3-hydroxykynurenine (3-HK) have the ability to cross cell membranes and penetrate tissue readily, or to cross the blood-brain and placental barriers. This is an important issue in view of the activity of the kynurenine pathway metabolites quinolinic acid and kynurenic acid noted above. Skorobogatov et al. have analysed the results of studies directly comparing blood and CNS levels (Cerebrospinal fluid or brain tissue) of kynurenine pathway compounds (**Figure 1**) or their relationship to symptoms and severity in a range of psychiatric conditions This demonstration should help in the interpretation of peripheral measurements in relation to disease progression and response to treatment for some psychiatric disorders. However, it is important to highlight that this may not be applicable to all disorders. For example, conditions with different degrees of involvement of the immune system, other peripheral tissues, and CNS may exhibit major differences because there is a differential production or modulation of kynurenine pathway activity influencing one tissue more than another.

**Figure 1:**
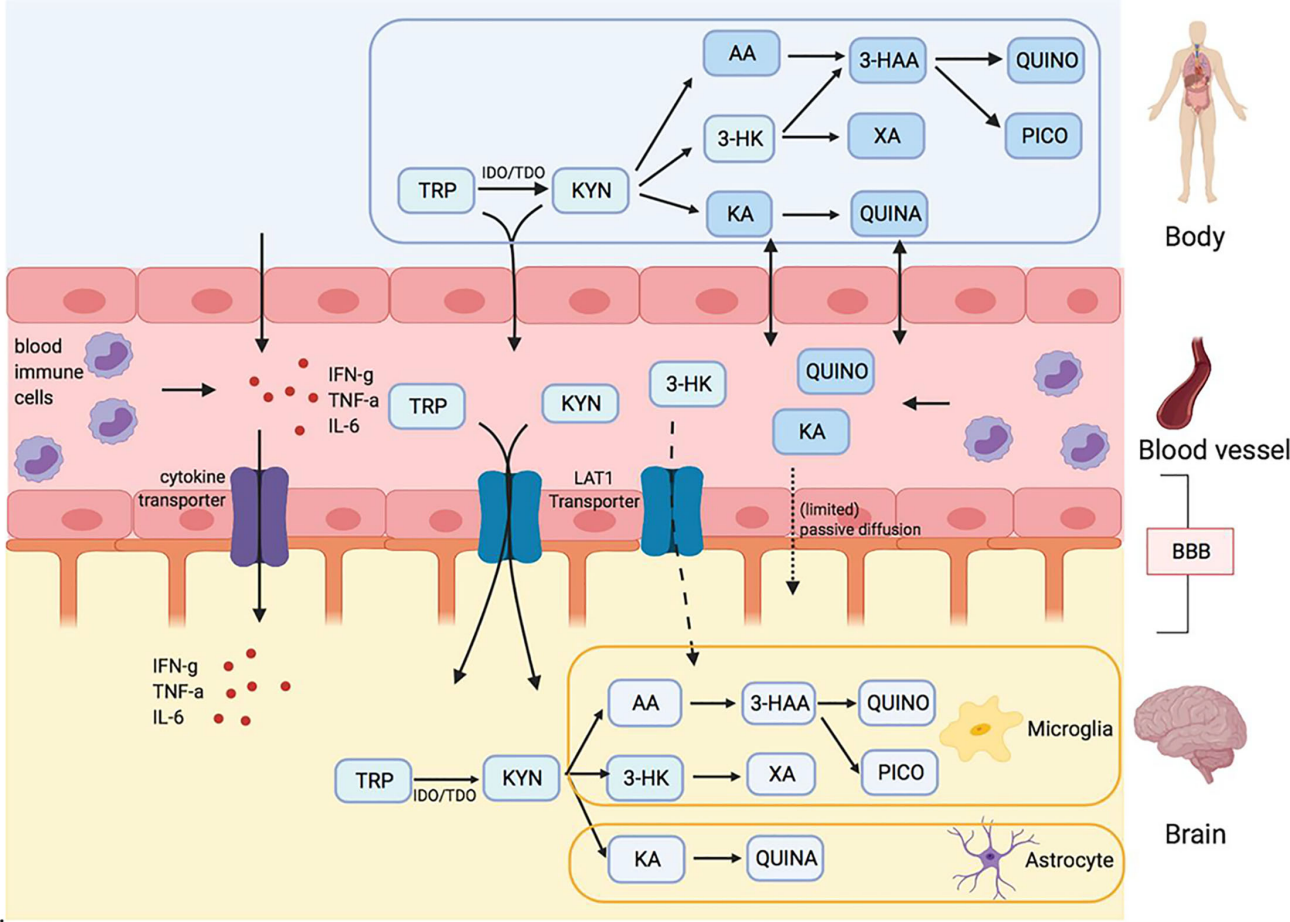
Kynurenine metabolites and the blood brain barrier (BBB) Skorobogatov et al. Tryptophan (TRP) and kynurenine (KYN), and to a lesser degree 3-hydrox kynurenine (3-HK) are actively transported into the brain over LAT1 transporters. Downstream metabolites of the kynurenine pathway (KP), like quinolinic acid (QUINO) and kynurenic acid (KA), cannot make use of these transporters, but (probably limited) passive diffusion of these metabolites over the BBB is possible. Anthranilic acid AA and 3-hydroxy anthranilic acid (not shown in figure) may equally pass the blood brain barrier through passive diffusion, much like QUINO. In the brain, microglia are responsible for the production of metabolites 3-HK and QUINO, whereas astrocytes produce KA. Peripheral production of these KP metabolites is done by blood immune cells, such as blood monocytes (PBMC) and other organs, including liver and kidney. The gut microbiome, which plays a role in psychiatric illness through the gut-brain axis, also affects KP metabolization.

Hebbrecht et al. summarise a systematic review of 21 studies of patients with bipolar disorder. The conclusion was drawn that kynurenine pathway activity is lower in these patients than in control subjects. This is an important outcome, as a large body of literature has suggested increased kynurenine pathway activity in major depressive disorders. The qualitative difference in the results may therefore provide valuable clues as to the aetiology, symptomatic differences, long-term prognosis, and treatment options in these rather different disorders.

ALS (Amyotrophic Lateral Sclerosis) is a late onset neurodegenerative disease. Neuroinflammation and the kynurenine pathway have been functionally implicated in many neurodegenerative diseases including ALS (11). Fifita et al. investigated the genetic contribution of 18 genes involved in tryptophan metabolism in patients with sporadic ALS. They concluded that genetic variation in four genes were directly involved in kynurenic acid synthesis from 3-hydroxykynurenine, and these genes may be associated with sporadic ALS and may confer risk to developing disease.

Mondanelli et al. evaluated the *in vivo* IL-6 dependency of IDO1 expression and activity in obesity. A dominant role of IL-6 in upregulating IDO1 in the adipose tissue of obese mice was observed.

We would like to thank all the contributors to this Research Topic and the reviewers for generously giving their time and expertise.

## Author Contributions

YM, TS, GG, performed a significant work in composition of the Editorial, LV and RW further supported it with their notices.

## Funding

YM: GINOP 2.3.2-2015-16-00034. RW: Medical Research Council, Biology and Biotechnology Research Council, Epsom Medical Research. LV: MTA-ELKH-SZTE Neuroscience Research Group and OTKA-138125-K.

## Conflict of Interest

The authors declare that the research was conducted in the absence of any commercial or financial relationships that could be construed as a potential conflict of interest.

## Publisher’s Note

All claims expressed in this article are solely those of the authors and do not necessarily represent those of their affiliated organizations, or those of the publisher, the editors and the reviewers. Any product that may be evaluated in this article, or claim that may be made by its manufacturer, is not guaranteed or endorsed by the publisher.
